# Food allergen sensitization pattern in adults in relation to severity of atopic dermatitis

**DOI:** 10.1186/2045-7022-4-9

**Published:** 2014-03-28

**Authors:** Heike Röckmann, Maartje J van Geel, Andre C Knulst, Jorike Huiskes, Carla AFM Bruijnzeel-Koomen, Marjolein S de Bruin-Weller

**Affiliations:** 1 Department of Dermatology/Allergology, University Medical Center Utrecht, Heidelberglaan 100, G 02.124, 3584 Utrecht, CX, The Netherlands; 2 Currently at Department of Dermatology, Radboud University Nijmegen Medical Center, Nijmegen, The Netherlands

**Keywords:** Atopic dermatitis, Component-resolved diagnostics, IgE food sensitization, Multiplexed allergen microarray

## Abstract

**Background:**

Limited data are available on the frequency of IgE mediated food sensitization and food allergy (FA) in adults with atopic dermatitis (AD).

**Objective:**

We investigated the pattern of food sensitization in adults with AD in relation to AD severity using multiplexed allergen microarray.

**Methods:**

211 adult patients referred between January 2010-July 2011 for evaluation of AD were unselectively included. Severity of AD was determined by therapy intensity, SASSAD-skin-score and sTARC levels. Allergen specific sIgE levels were measured by ImmunoCAP ISAC® microarray. FA was defined as convincing history taken by physician and sensitization to the corresponding allergen.

**Results:**

Sensitization to food was found in 74.4% of the AD patients, 54% had a positive history of FA and 20.4% asymptomatic sensitization. There was no association between severity of AD and frequency of food sensitization or history of FA.

Sensitization to PR-10 related food allergens occurred most frequently (63.5%) and was independent from AD severity. Correspondingly, pollen-food syndrome accounted for most of the FA, being also independent from AD severity. Of all plant food allergens only sensitization to *nAra h 1* was significantly more frequent in patients with severe AD. In the total group 75 (35.5%) patients with AD showed sensitization to any animal food allergen. The percentage was significantly higher in patients with severe AD (51.4%) compared to patients with mild/moderate AD (27.7%). Sensitization to cow’s milk allergens, in particular to *nBos d lactoferrin*, was more frequent in severe AD patients.

**Conclusion:**

AD was frequently associated with food sensitization. The percentage of sensitization to animal food allergens was significantly higher in severe AD patients.

## Background

Atopic dermatitis (AD) is a chronic inflammatory skin disease that commonly begins in early infancy and runs a course of exacerbations and remissions. AD is often accompanied by other atopic features such as asthma, allergic rhino conjunctivitis and food allergy [1,2]. AD affects 1-3% of adults world-wide [3]; the prevalence of immediate type FA in adults is 2-3% [4]. In infants with AD the prevalence of immediate type FA was estimated to be 30% [2,5,6]. So far data on the frequency of immediate type FA in adult patients with AD are scarce and show a wide variation (33% versus 73%) dependent on study design [7-9]. In addition, there are no data on the association between severity of AD in adults and food sensitization pattern.

Allergen specific IgE sensitization (symptomatic as well as asymptomatic) to a wide spectrum of food-and aeroallergens is a common phenomenon in adult patients with AD; especially sensitization to house dust mite, pollen and (pollen-related cross-reactive) food allergens is frequently observed [2,7,9,10]. Food sensitization in an unselected group of adult AD patients using component resolved diagnostics was only investigated in a small German population (n = 20) [11] describing a sensitization frequency of 70% to PR-10 related food allergens, 45% to animal food and 30% to CM allergens. However, comparison of patients depended on AD severity was not done.

Routine tools for detection of sensitization are in-vivo skin prick testing (SPT) with allergens or in-vitro determination of allergen specific IgE in serum by ImmunoCAP® [12]. The newly developed microarray (ImmunoCap ISAC®) incorporates recombinant or purified allergen components from more than 50 allergenic sources, allowing determination of specific immunoglobulin E (IgE) against currently more than 130 specific allergen components from the same serum sample [13-16]. Notably, measuring allergen specific IgE only detects sensitization. Until now, no data are available on the relation between the pattern of IgE mediated food sensitisation and severity of AD in adults. In this retrospective study we investigated the frequency and type of food sensitization in adult patients with AD in relation to AD severity.

## Methods

All patients (aged ≥16 years) diagnosed with AD who visited the outpatient clinic of the department of Dermatology/Allergology of the University Medical Center Utrecht between January 2010- July 2011 were retrospectively analyzed. All patients were primary referred to our hospital for evaluation and treatment of AD. Inclusion criteria were: reference due to evaluation of AD, diagnosis of AD, clinical data about AD therapy, specific IgE determined by ImmunoCap ISAC® microarray. Exclusion criteria were: uncertainty about AD diagnosis. Data on AD (present and previous therapy; SASSAD [17] [Six Area, Six Sign Atopic Dermatitis] skin score, sTARC [18] [serum thymus and activation-regulated chemokine], FA in patient history (type of food, symptoms, severity of reaction) and other atopic features (asthma, seasonal allergic rhino conjunctivitis, allergy to animal dander) were obtained from patient records.

The medical ethics committee of University Medical Center Utrecht confirmed that the Medical Research Involving Human Subjects Act (WMO) does not apply to the study.

### Atopic dermatitis

Diagnosis of AD was made according to the UK criteria of Williams [19]. Severity of AD was defined based on the therapy the patients received to control their disease. Patients using topical steroids to control their disease were defined having mild/moderate AD. Patients who were prescribed oral immunosuppressive drugs continuously for more than 3 months in the previous 5 years were defined as having severe AD. Clinical eczema severity scores (SASSAD; severity range 0 to108), serum total IgE and sTARC at patient’s first visit to the outpatient clinic were available in a subgroup and used for the confirmation of the classification based on therapy. Concentrations of sTARC were determined using the Human TARC Immunoassay which was performed according to the manufacturer’s instructions (R&D systems, Minneapolis, USA).

### IgE measurement

Specific IgE levels were routinely measured using a microarray immunoassay (Immuno Solid-phase allergen Chip (ImmunoCAP ISAC®, VBC Genomics and Phadia) with 103 allergens, (see Additional file 1: Table S1 in the Online Repository) in every adult patient with AD visiting the outpatient clinic from January 2010 to July 2011. A value of < 0.3 ISU/L was considered negative; sensitization was defined as at least one food allergen on the ISAC® > 0.3 ISU/L. Median serum level and interquartile range (IQR) was assessed in patients with values ≥ 0.3 ISU/L.

Total IgE levels were routinely measured using ImmunoCAP (Phadia®). Levels > 5000 kU/l were not further titrated.

### Food allergy

FA was defined as a convincing history (taken by an experienced physician) of immediate type symptoms (oral allergy symptoms, urticaria, angioedema, conjunctivitis, rhinitis, vomiting, diarrhea, dyspnea, hypotension, anaphylactic shock) within 2 hours after intake of the culprit food in combination with sensitization to at least one corresponding allergen on the ISAC®.

Considering each food item separately, severity of the FA-related symptoms according to history (taking into account the most severe reaction) was classified according to a modification of the Mueller classification [20] (grade 0-4, grade 4 most severe).

Diagnosis of asymptomatic sensitization was made in sensitized AD patients who stated that they tolerated all foods.

### Atopic comorbidity

The presence of seasonal allergic rhino conjunctivitis (AR), asthma (AA) and inhalation allergy to animal dander was registered in order to assess the role of pollen-food cross reactivity. Diagnosis of asthma (AA), was based on two criteria: diagnosis made by a physician (general practitioner or pulmonologist) and/or daily use of inhalant corticosteroids. Diagnosis of seasonal allergic rhino conjunctivitis (AR) and allergy to animal dander was based on patient history and sensitization to the corresponding inhalant allergen on the ISAC®.

### Statistical analysis

Statistical analysis was performed using software (SPSS for Windows, version 19, SPSS Inc, Chicago, IL). Associations between variables were tested in contingency tables using Chi-square tests. For skewed non-categorical data we used Mann–Whitney test. IgE measurement using a microarray immunoassay is sensitive for a false discovery rate by multiple testing. Therefore, adjusted p-value < 0.01 were considered to be statistically significant. Due to the hypothetical phase of this study no Bonferroni–correction was applied. All comparisons were made two-tailed.

## Results

### Patient characteristics

In total 211 patients referred for evaluation of AD were included. According to the earlier defined criteria, 141 of 211 patients (66.8%) had mild/moderate AD and 70 patients (33.2%) suffered from severe AD. Patient characteristics are shown in Table 1.

**Table 1:** Patient characteristics

	**Total n = 211**	**Mild/moderate AD n = 141**	**Severe AD n = 70**	**P-value**^**†**^
**Female, n (%)**^**†**^	138 (65.4)	102 (72.3)	36 (51.4)	0.003*
**Age, year**^**1,2**^	28.0 (22.0-44.0)	26.0 (21.0-42.0)	39.0 (26.0-49.0)	< 0.001*
**Total IgE, kU/L**^**1,2,3**^	1455.0 (372.5-5000)	694 (171-2099)	5000 (1884.5-5000)	< 0.001*
**TARC, pg/mL**^**1,2,4**^	1328.0 (470.0-3908.0)	743 (336.3-1683.3)	2826 (1682-7819)	< 0.001*
**SASSAD**^**1,2,4**^	21.0 (10.0-31.0)	17.0 (8.0-27.8)	24.0 (18.0-38.0)	< 0.001*
**Other atopic diseases:**				
**Asthma,**^**5**^^**† **^**n (%)**	102 (49.8)	68 (49.3)	34 (50.7)	0.843
**AR, seasonal, tree/grass,**^**6**^^**† **^**n (%)**	131 (64.9)	88 (64.2)	43 (66.2)	0.789
**AR, cat/dog,**^**7**^^**† **^**n (%)**	100 (55.2)	67 (53.6)	33 (58.9)	0.505

^1^Median (IQR); ^2^p < 0.001; Mann–Whitney U-test; ^3^In subgroups mild/moderate (n = 65) versus severe AD (n = 36); ^4^In subgroups mild/moderate (n = 92) versus severe AD (n = 51); ^5^Unknown in n = 6; ^6^Unknown in n = 9; ^7^Unknown in n = 30; *p < 0.01.

^†^Chi-square; mild/moderate versus severe AD.

The median SASSAD score and sTARC (subgroup n = 143) was 21 and 1328 pg/ml respectively. This was 17 and 743 pg/ml respectively in a subgroup of mild/moderate AD patients (n = 92) and significantly lower compared to the median SASSAD score of 24 and sTARC of 2826 pg/ml in a subgroup of severe AD patients (n = 51) (p < 0.001). Table 1 shows that also total serum-IgE levels were significantly increased in the group of patients with severe AD (p < 0.001).

Patients with severe AD had a significantly higher age and were significant more often men compared to patients with mild/moderate AD (Table 1).

Distribution of asthma, seasonal allergic rhino conjunctivitis and allergy to dog and/or cat was not significantly different between patients with mild/moderate and severe AD.

### Food sensitization and positive history of food allergy (frequency and severity) in adult patients with atopic dermatitis

In the total group of patients with AD food sensitization was found in 74,4%, 54% had a positive history of FA and 20.4% asymptomatic food sensitization (tolerant to all foods). These percentages were not significantly different between the group with mild/moderate and the group with severe AD (Table 2).

**Table 2:** Proportion positive history of FA and asymptomatic sensitization to food allergens in patients with AD (mild/moderate versus severe AD)

**Group analysis**	**Total n = 211 (%)**	**Mild/moderate AD n = 141 (%)**	**Severe AD n = 70 (%)**
**AD; no FA, no sensitization**	54 (25.6)	41 (29.1)	13 (18.6)
**AD; no FA, only sensitization**	43 (20.4)	27 (19.1)	16 (22.9)
**AD; FA supported by sensitization**	114 (54.0)	73 (51.8)	41 (58.6)

p–value = 0.26; Chi-square; mild/moderate versus severe AD.

Comparison of AD patients with a positive history of FA to AD patients with asymptomatic sensitization did not show differences in age, sex, sTARC or total serum IgE. In both groups sensitization to PR-10 related food allergens was most frequent.

No significant difference in severity of FA was found between patients with mild/moderate and severe AD. Mueller 3 or 4 reactions occurred in 41.1% and 46.3% of the patients with respectively mild/moderate or severe AD (see Additional file 2: Table S2 in the Online Repository).

### Sensitization to food allergens of plant origin

Sensitization to any plant food allergen available in the ISAC® microarray was found in 150 of 211 AD patients (71.1%). Sensitization to any PR-10 related plant food allergen available in the ISAC® microarray was seen most frequently in 134 of 211 AD patients (63.5%) without statistical difference between mild/moderate and severe AD. We observed a significantly (p = 0.001) higher percentage of sensitization to the peanut allergen *nAra h 1* in severe AD patients compared to mild/moderate AD patients (Table 3).

**Table 3:** Sensitization (frequency and serum level) to food allergens of plant origin: selection of food allergens most relevant in the Dutch population

**Food**	**Allergen**		**Total n = 211**	**Mild/moderate AD n = 141**	**Severe AD n = 70**	**P-value n (%)**^**1 **^ ***median***^***2***^
**Kiwi**		n (%)	76 (36.0)	44 (31.2)	32 (45.7)	0.04
	nAct d 1	n (%)	24 (11.4)	11 (7.8)	13 (18.6)	0.02
	*(cyst. protease)*	*Median (IQR)*	*1.83 (0.49-10.95)*	*2.10 (1.06-11.32)*	*0.71 (0.36-13.39)*	*0.28*
	nAct d 2	n (%)	25 (11.8)	17 (12.1)	8 (11.4)	0.89
	*(TLP)*	*Median (IQR)*	*1.92 (1.08-3.23)*	*1.92 (1.49-3.44)*	*1.36 (0.82-3.19)*	*0.29*
	nAct d 5	n (%)	1 (0.5)	0 (0)	1 (1.4)	n.r.
	*(Kiwellin)*	*Median (IQR)*	*0.62 (0.62-0.62)*	*-*	*0.62 (0.62-0.62)*	*n.r.*
	nAct d 8	n (%)	44 (20.9)	27 (19.1)	17 (24.3)	0.39
	*(PR-10)*	*Median (IQR)*	*1.93 (0.76-7.17)*	*1.83 (0.74-3.93)*	*2.52 (1.12-10.81)*	*0.16*
**Peanut**		n (%)	119 (56.4)	74 (52.5)	45 (64.3)	0.10
	nAra h 1	n (%)	24 (11.4)	9 (6.4)	15 (21.4)	0.001*
	*(SP)*	*Median (IQR)*	*6.78 (1.45-11.83)*	*7.70 (3.93-17.16)*	*4.62 (0.95-12.09)*	*0.46*
	nAra h 2	n (%)	23 (10.9)	14 (9.9)	9 (12.9)	0.52
	*(SP)*	*Median (IQR)*	*15.78 (5.55-35.6)*	*11.41 (4.89-33.30)*	*32.69 (8.18-56.05)*	*0.31*
	nAra h 3	n (%)	16 (7.6)	7 (5.0)	9 (12.9)	0.04
	*(SP)*	*Median (IQR)*	*4.82 (1.0-22.66)*	*1.61 (0.88-42.95)*	*5.60 (1.33-22.26)*	*0.79*
	rAra h 8	n (%)	112 (53.1)	70 (49.6)	42 (60.0)	0.16
	*(PR-10)*	*Median (IQR)*	*4.73 (1.66-13.02)*	*3.76 (1.57-9.18)*	*8.81 (1.85-16.16)*	*0.08*
**Hazelnut**		n (%)	120 (56.9)	78 (55.3)	42 (60.0)	0.52
	rCor a 1.0401	n (%)	117 (55.5)	76 (53.9)	41 (58.6)	0.52
	*(PR-10)*	*Median (IQR)*	*5.29 (2.30-12.37)*	*4.76 (2.18-12.0)*	*5.63 (2.38-16.03)*	*0.50*
	rCor a 8	n (%)	11 (5.2)	7 (5.0)	4 (5.7)	n.r.
	*(LTP)*	*Median (IQR)*	*2.78 (0.50-5.49)*	*2.94 (0.85-5.49)*	*0.81 (0.37-28.58)*	*0.35*
	nCor a 9	n (%)	12 (5.7)	9 (6.4)	3 (4.3)	n.r.
	*(SP)*	*Median (IQR)*	*1.49 (0.69-1.60)*	*1.50 (0.98-1.60)*	*0.60 (0.49-x)*	*0.64*
**Apple**	rMal d 1	n (%)	126 (59.7)	81 (57.4)	45 (64.3)	0.34
	*(PR-10)*	*Median (IQR)*	*12.35 (4.50-36.50)*	*10.00 (3.76-29.2)*	*24.02 (5.35-51.38)*	*0.06*
**Peach**		n (%)	122 (57.8)	81 (57.4)	41 (58.6)	0.88
	rPru p 1	n (%)	121 (57.3)	80 (56.7)	41 (58.6)	0.80
	*(PR-10)*	*Median (IQR)*	*9.08 (2.73-20.78)*	*6.85 (2.33-13.11)*	*11.00 (4.18-30.61)*	*0.02*
	nPru p 3	n (%)	11 (5.2)	8 (5.7)	3 (4.3)	n.r.
	*(LTP)*	*Median (IQR)*	*4.40 (2.02-7.21)*	*4.70 (1.88-6.95)*	*2.70 (2.02-x)*	*> 0.99*
**Tree PR-10**		n (%)	137 (64.9)	91 (64.5)	46 (65.7)	0.87
	rBet v 1	n (%)	136 (64.5)	90 (63.8)	46 (65.7)	0.79
		*Median (IQR)*	*34.97 (11.29-80.86)*	*32.95 (9.36-57.11)*	*48.5 (17.86-116.25)*	0.009*
	rAln g 1	n (%)	129 (61.1)	84 (59.6)	45 (64.3)	0.51
		*Median (IQR)*	*13.52 (3.37-31.13)*	*10.25 (3.21-29.70)*	*14.89 (4.42-32.04)*	*0.33*
	rCor a 1.0101	n (%)	118 (55.9)	76 (53.9)	42 (60.0)	0.40
		*Median (IQR)*	*7.00 (2.45-20.83)*	*7.35 (3.14-20.57)*	*12.50 (6.09 -22.90)*	*0.13*
**Total PR-10**		n (%)	134 (63.5)	88 (62.4)	46 (65.7)	0.64

^1^Chi-square; mild/moderate versus severe AD; ^2^Mann–Whitney U-test; mild/moderate versus severe AD. *p < 0.01; *n.r,* non-reliable, due to small number; PR-10 protein Bet v1 homulogus; SP: storage protein; LTP: lipid transfer protein; TLP: Thaumatin-like protein.

No differences were found concerning sensitization to *nAra h 2, nAra h 3* and *rAra h 8* or one of the other plant food allergens available in the ISAC® microarray (see Additional file 3: Table S3 in the Online Repository).

Median serum level of IgE antibodies to *nAra h 1* or other food allergens did not differ between patients with severe AD compared to patients with mild/moderate AD (Table 3).

Table 4 shows the number of patients with a positive history of immediate type FA to a selection of plant food allergens most relevant for the Dutch population. Percentages of a positive history of FA vary dependent on the specific food item between 12.1% for peach and 34.8% for apple. No differences in a positive history of FA to any plant food were found between patients with mild/moderate AD and severe AD.

**Table 4:** Frequency of allergy to food allergens of plant origin: selection of foods most relevant in the Dutch population

**Food n (%)**	**Total n = 211**	**Mild/moderate AD n = 141**	**Severe AD n = 70**	**P-value**^**1**^
**Kiwi**	37 (17.5)	25 (17.7)	12 (17.1)	0.92
**Peanut**	43 (20.4)	27 (19.1)	16 (22.9)	0.53
**Hazelnut**	35 (16.6)	24 (17.0)	11 (15.7)	0.81
**Apple**	70 (33.2)	49 (34.8)	21 (30.0)	0.49
**Peach**	28 (13.3)	17 (12.1)	11 (15.7)	0.46

^1^Chi-square; Mild/moderate versus severe AD.

Allergy based on positive patient history.

Frequency of sensitization to one or more of the *PR-10* related *inhalant allergens* (*rAln g 1, rBet v 1, rCor a 1.0101*) occurred frequently (64.9%) but not different in patients with severe AD compared to mild/moderate AD (Table 3). A significant (*p-value = 0.009*) higher median IgE serum level against *rBet v 1* was found in patients with severe AD compared to mild/moderate AD.

### Sensitization to food allergens of animal origin

In the total group of patients with AD 75 patients (35.5%) showed sensitization to any animal food allergen available in the ISAC® microarray (Table 5). Sensitization to any food allergen of animal origin was found in of 51.4% patients with severe AD. This was significantly (p = 0.001) higher compared to the group with mild/moderate AD (27.7%).

**Table 5:** Sensitization (frequency and serum level) to food allergens of animal origin

**Food allergen**			**Total n = 211**	**Mild/moderate AD n = 141**	**Severe AD n = 70**	**P-value n (%)**^**1 **^ ***median***^***2***^
**Cow’s milk**		n (%)	51 (24.2)	26 (18.4)	25 (35.7)	0.006*
	nBos d 4	n (%)	10 (4.7)	8 (5.7)	2 (2.9)	n.r.
	*(α-lactalbu.)*	*Median (IQR)*	*0.54 (0.32-2.63)*	*0.54 (0.31-2.37)*	*1.60 (0.40-x)*	*0.60*
	nBos d 5	n (%)	10 (4.7)	6 (4.3)	4 (5.7)	n.r.
	*(β-lactoglob.)*	*Median (IQR)*	*1.44 (0.71-4.21)*	*0.89 (0.47-3.04)*	*2.59 (1.98-11.67)*	*0.06*
	*nBos d 6*	n (%)	11 (5.2)	6 (4.3)	5 (7.1)	n.r.
	*(serum albu.)*	*Median (IQR)*	*1.01 (0.53-4.04)*	*0.81 (0.52-5.58)*	*1.54 (0.59-5.89)*	*0.72*
	*nBos d 8*	n (%)	25 (11.8)	14 (9.9)	11 (15.7)	0.22
	*(casein)*	*Median (IQR)*	*2.23 (1.11-5.89)*	*1.73 (0.80-4.75)*	*3.30 (1.27-8.69)*	*0.30*
	*nBos d lactoferrin*	n (%)	34 (16.1)	16 (11.3)	18 (25.7)	0.008*
	*(transferrin)*	*Median (IQR)*	*1.42 (0.68-2.45)*	*0.97 (0.50-2.05)*	*1.95 (1.12-2.92)*	*0.05*
**Hen’s egg**		n (%)	28 (13.3)	16 (11.3)	12 (17.1)	0.24
	nGal d 1	n (%)	23 (10.9)	12 (8.5)	11 (15.7)	0.11
	*(ovomucoid)*	*Median (IQR)*	*1.96 (0.63-5.19)*	*2.84 (1.24-5.36)*	*1.16 (0.46-5.19)*	*0.24*
	nGal d 2	n (%)	10 (4.7)	4 (2.8)	6 (8.6)	n.r.
	*(ovalbumin)*	*Median (IQR)*	*1.64 (0.51-3.64)*	*0.71 (0.42-4.69)*	*2.21 (1.05-6.63)*	*0.29*
	nGal d 3	n (%)	6 (2.8)	3 (2.1)	3 (4.3)	n.r.
	*(ovotransfer.)*	*Median (IQR)*	*1.08 (0.68-2.68)*	*0.78 (0.69-x)*	*2.04 (0.63-x)*	*0.51*
	nGal d 5	n (%)	4 (1.9)	0 (0)	4 (5.7)	n.r.
	*(serum albu.)*	*Median (IQR)*	*0.43 (0.31-094)*	-	*0.43 (0.31-094)*	-
**Fish**		n (%)	24 (11.4)	12 (8.5)	12 (17.1)	0.06
	rCyp c 1	n (%)	23 (10.9)	12 (8.5)	11 (15.7)	0.11
	*(parvalb.)*	*Median (IQR)*	*2.43 (0.50-15.0)*	*2.00 (0.45-8.80)*	*2.43 (0.50-21.78)*	*0.30*
	rGad c 1	n (%)	13 (6.2)	5 (3.5)	8 (11.4)	n.r.
	*(parvalb.)*	*Median (IQR)*	*4.96 (2.47-7.85)*	*5.80 (4.65-7.85)*	*3.71 (0.93-18.72)*	*0.24*
**Shrimp**		n (%)	15 (7.1)	5 (3.5)	10 (14.3)	n.r.
	rPen a 1	n (%)	8 (3.8)	2 (1.4)	6 (8.6)	n.r.
	*(tropomyosin)*	*Median (IQR)*	*10.18 (1.24-33.22)*	*28.44 (18.87-x)*	*3.85 (0.54-37.20)*	*0.18*
	nPen i 1	n (%)	11 (5.2)	4 (2.8)	7 (10.0)	n.r.
	*(tropomyosin)*	*Median (IQR)*	*5.33 (1.50-26.0)*	*11.08 (0.91-40.31)*	*5.33 (1.50-26.0)*	*0.85*
	nPen m 1	n(%)	13 (6.2)	4 (2.8)	9 (12.9)	n.r.
	*(tropomyosin)*	*Median (IQR)*	*5.14 (0.48-30.46)*	*11.24 (0.39-43.73)*	*5.14 (0.70-23.89)*	*> 0.99*
**Total**^**3**^		n (%)	75 (35.5)	39 (27.7)	36 (51.4)	0.001*

^1^Chi-square; mild/moderate versus severe AD. *p < 0.01.; ^2^Mann–Whitney U-test; mild/moderate versus severe AD.

^3^Frequency of sensitization to any of the animal allergens. *n.r,* non-reliable, due to small number.

Most frequently (24.2%) sensitization to cow’s milk (CM) allergens was detected. Frequency of sensitization to CM allergens differed significantly (p = 0.006) between mild/moderate and severe AD patients (18.4% versus 35.7%). Analysis of sensitization to CM components showed that most frequently sensitization to *nBos d lactoferrin* (16.1%) and *nBos d 8* (11.8%) occurred in patients with AD. Patients with severe AD were significantly more frequently sensitized to *nBos d lactoferrin* than patients with mild/moderate AD. Median serum levels of antibodies to CM components tended to be higher in severe AD patients without statistical significance according to a p-value < 0.01 (Table 5). No valid statistical analysis could be performed regarding *nBos d 4, nBos d 5* and *nBos d 6* due to low patient numbers*.*

Frequency and median serum levels of sensitization to hen’s egg, fish and shrimp is shown in Table 5. No difference between mild/moderate and severe AD patients has been observed.

A positive history of immediate type FA to animal foods assessed by patients history is presented in Table 6. In severe AD a positive history of FA to all animal foods tended to be more frequent (17.1%) than in patients with mild/moderate AD (10.6%). A positive history of FA to CM occurred most frequently (8.5%).

**Table 6:** Frequency of allergy to food allergens of animal origin

**Allergen**	**Total**	**Mild/moderate AD**	**Severe AD**	**P-value**^**1**^
	**n = 211 (%)**	**n = 141 (%)**	**n = 70 (%)**	
Cow’s milk	18 (8.5)	10 (7.1)	8 (11.4)	0.29
Hen’s egg	12 (5.7)	11 (7.8)	1 (1.4)	n.r
Fish	8 (3.8)	4 (2.8)	4 (5.7)	n.r
Shrimp	4 (1.9)	2 (1.4)	2 (2.9)	n.r.
**Total**^**2**^	27 (12.8)	15 (10.6)	12 (17.1)	0.18

^1^Chi-square; mild/moderate AD versus severe AD.

^2^Allergy to cow’s milk *or* hen’s egg *or* fish *or* shrimp.

*n.r,* non-reliable, due to small number.

Allergy based on positive patient history.

CM allergy was seen in 11.4% of severe AD patients, while only in 7.1% of mild/ moderate AD patients. No valid statistical analysis could be performed regarding hen’s egg, fish and shrimp due to low patient numbers*.*

50% of all CMA patients with AD reported severe symptoms (Mueller grade 3 or 4).

## Discussion

To our knowledge, this is the first study analyzing the pattern of food allergen sensitization using the multiplexed allergen microarray in adults in relation to severity of AD.

We differentiated severity of AD based on the type of therapy patients received to control their disease (local versus systemic) and confirmed this classification by clinical skin scores (SASSAD) and sTARC levels. Notably, patients with severe AD had a significantly higher age than patients with mild/moderate AD, possibly due to a larger proportion of patients with chronic severe AD in our third line outpatient clinic.

Sensitization to food was found in 74.4% of all AD patients, 54% had a positive history of FA and 20.4% of all patients had asymptomatic sensitization. Sensitization to specific foods occurred two to three times more frequent than a positive history of food allergy to the respective food allergen. This underlines the high risk of false positive results by unselective in-vitro testing of food sensitization.

There was no difference in serum levels of any specific common food or inhalant allergen (apart from Bet v 1, which was only moderate). Therefore, the considerable difference of total IgE between mild/moderate and severe AD, cannot be explained by specific IgE sensitization. Remarkably, there was no association between severity of AD and frequency of food sensitization or a positive history of FA. Only one study in infants related AD severity, differentiated by duration of topical steroid usage, to the presence of food sensitization and FA. This study observed an increased frequency of sensitization to food (measured by SPT) and immediate type FA (parents reported) with increased AD severity [21].

The majority of studies on the prevalence of food sensitization in patients with AD were performed in children using SPT or CAP and show varying rates up to 86% [6,22-24]. A recent study [25] using ISAC® micro array in 140 children with AD (mean age 74 months) described IgE sensitization profiles without considering differences in AD severity. Sensitization to hen’s egg was found most commonly in 35%, followed by sensitization to apple (23%), hazelnut (22%), kiwi (15%) and CM (14%). Studies investigating the co-prevalence of AD and food sensitization or FA in adults are scarce and difficult to compare due to different study designs [7,9]. Only one small study [11] reported IgE profiling using ISAC® micro array in adult AD patients (n = 20), 70% (n = 14) of them with severe AD. Results show a sensitization frequency of 70% to PR-10 related food allergens, 45% to animal food and 30% to CM allergens, being in accordance with our data.

In our study, sensitization to PR-10 related plant food allergens were most frequent. This corresponded with a high frequency of seasonal rhino conjunctivitis. However, no difference in PR-10 related plant food allergen sensitization was seen between mild/moderate and severe AD patients. Analysis of the allergen sensitization pattern shows that this is most likely due to pollen cross-reactivity [26-28]. However, severe AD patients showed a higher sensitization rate to the plant allergen *nAra h 1* (major peanut allergen), which is not pollen cross-reactive [29]. However, there was no difference in *nAra h 1* median IgE serum level or history of immediate type FA to peanut. The study of Ott et al. [11] using ISAC® micro array reported only one patient (5%) in their adult AD population with sensitization to *nAra h 1* without further differentiation of AD severity. In children with AD a high rate of sensitization to peanut (varying between 33% en 86%), analyzed by SPT and/or CAP was reported [22-24]. Data specific for *nAra h 1* sensitization were not available*.*

In the total group of patients with AD 35.5% showed sensitization to any animal food allergen. The percentage was significantly higher in patients with severe AD (51.4%) compared to patients with mild/moderate AD (27.7%). However, no difference in serum levels of any animal allergen was observed. In severe AD patients, sensitization to the CM allergens (35.7%), in particular to *nBos d lactoferrin* (25.7%), was observed more frequently compared to patients with mild/moderate AD (18.4% and 11.3% respectively). A comparable rate of sensitization to animal food and CM measured by ISAC® microarray was shown previously in a small population [11] (n = 20) of adult AD patients without differentiation of severity. Notably, in this study the microarray did not contain *lactoferrin* and sensitization to *nBos d 6* was seen most frequently (30%). This is different from our results, where sensitization to *nBos d 6* was found in 5.2% of the population.

Frequency of a positive history of FA to all animal food was 12.8% and for CM 8.5%. Both tended to be more frequent in severe AD patients (17.1% and 11.4%, respectively). In adults we previously described a frequent co-prevalence of AD and CM allergy [30]. A recent observational study by Wood et al. [31] on children (n = 244; age 3-15 month) showed a much higher rate of resolution of CM allergy in infants with mild compared to severe AD, indicating that moderate to severe AD in childhood is highly predictive of persistent CM allergy.

## Conclusion

In conclusion, our results show that in this Dutch population of adult patients with AD sensitization to PR-10 related plant food allergens occurred most frequently, but independent from AD severity. In contrast, we found a higher sensitization rate to animal food, in particular CM in severe AD in adults. These findings need to be reproduced in a prospective study.

### Limitations

The retrospective design of this study is sensitive to missing data and may result in an underestimation of current frequency of FA. Since all patients have been referred for evaluation of AD, FA was not proven by food challenge but patient history by an experienced physician. Another limitation may be the recruitment of this population from a tertiary university center, which possibly results in a certain degree of selection bias.

At present there are limited data on the sensitivity and specificity of the separate allergen components of the ISAC® microarray compared to conventional methods like CAP.

Finally, the results might be influenced by geographical factors such as pollen exposure and eating habits and can possibly not generalized to al AD patient populations.

## Abbreviations

AD: Atopic dermatitis; FA: Food allergy; IgE: Immunoglobulin E; SPT: Skin prick test; ISAC: Immuno solid-phase allergen chip; TARC: Thymus and activation-regulated chemokine; SASSAD: Six Area, Six Sign Atopic Dermatitis; IQR: Interquartile range; AA: Asthma; AR: Allergic rhino conjunctivitis; n: Natural; r: Recombinant; CMA: Cow’s milk allergy; CM: Cow’s milk; LF: Lactoferrin; PR-10 protein: also called Bet v 1 homologue, belongs to the pathogenesis-related protein family PR-10 and shows IgE antibody cross-reactivity between homologous allergens in pollens and various fruits and vegetables.

## Competing interests

The authors declare that they have no competing interests.

## Authors’ contributions

MJG, JH, MSBW and HR were involved in the study design, data collection/-analysis/–interpretation and writing the manuscript. ACK and CAFMBK were involved in data interpretation and writing the manuscript. All authors read and approved the final manuscript.

## Supplementary Material

Additional file 1: Table S1 Allergens available on ImmunoCAP ISAC^®^ microarray. Click here for file

Additional file 2: Table S2 Severity of FA by Mueller classification in patients with AD (mild/moderate versus severe AD). Click here for file

Additional file 3: Table S3 Sensitization (frequency and serum level) to food allergens of plant origin (additional allergens). Click here for file
