# Supplementary material for: Food allergen sensitization pattern in adults in relation to severity of atopic dermatitis
Source: Clin Transl Allergy. 2014 Mar 28;4:9. doi: 10.1186/2045-7022-4-9 (PMC4022323; doi:10.1186/2045-7022-4-9)
Supplement: Additional file 1: Table S1 — Allergens available on ImmunoCAP ISAC® microarray. [file 2045-7022-4-9-S1.doc]

**Table S1 Allergens available on ImmunoCAP ISAC® microarray**

|  | **Allergen** |
| --- | --- |
| Foods of plant origin | nAct d 1 |
|  | nAct d 2 |
|  | nAct d 5 |
|  | rBer e 1 |
|  | rAna o 2 |
|  | nCor a 9 |
|  | nSes i 1 |
|  | nAra h 1 |
|  | nAra h 2 |
|  | nAra h 3 |
|  | nGly m 5 |
|  | nGly m 6 |
|  | nTri a 18 |
|  | nTri a Gliadin |
|  | rTri a 19.0101 |
|  | nTri a aA_TI |
| Grass pollen | nCyn d 1 |
|  | rPhl p 1 |
|  | rPhl p 2 |
|  | nPhl p 4 |
|  | rPhl p 5 |
|  | rPhl p 6 |
|  | rPhl p 11 |
| Tree pollenn | nOle e 1 |
|  | rPla a 1 |
|  | nPla a 2 |
|  | nCry j 1 |
|  | nCup a 1 |
| Weed pollen | nAmb a 1 |
|  | nArt v 1 |
|  | nSal k 1 |
| Latex | rHev b 1 |
|  | rHev b 3 |
|  | rHev b 5 |
|  | rHev b 6 |
| PR-10 | rBet v 1 |
|  | rAln g 1 |
|  | rCor a 1.0101 |
|  | rCor a 1.0401 |
|  | rMal d 1 |
|  | rPru p 1 |
|  | rGly m 4 |
|  | rAra h 8 |
|  | nAct d 8 |
|  | rApi g 1 |
|  | rDau c 1 |
| nsLTP | nPru p 3 |
|  | rCor a 8 |
|  | nArt v 3 |
|  | rPar j 2 |
| Profilin | rBet v 2 |
|  | nOle e 2 |
|  | rHev b 8 |
|  | rMer a 1 |
|  | rPhl p 12 |

|  | **Allergen** |
| --- | --- |
| CA binding | rBet v 4 |
|  | rPhl p 7 |
| CCD | nAna c 2 |
| Foods of animal origin | nGal d 1 |
|  | nGal d 2 |
|  | nGal d 3 |
|  | nGal d 5 |
|  | nBos d 4 |
|  | nBos d 5 |
|  | nBos d 8 |
|  | nBos d lactoferrin |
| Cock-roach | rBla g 1 |
|  | rBla g 2 |
|  | rBla g 4 |
|  | rBla g 5 |
| Animal | rFel d 1 |
|  | rFel d 4 |
|  | rCan f 1 |
|  | rCan f 2 |
|  | nMus m 1 |
| Mould | rAlt a 1 |
|  | rAlt a 6 |
|  | rAsp f 1 |
|  | rAsp f 2 |
|  | rAsp f 3 |
|  | rAsp f 4 |
|  | rAsp f 6 |
|  | rCla h 8 |
| Parasite | rAni s 1 |
| Venom | nApi m 1 |
|  | nApi m 4 |
| Mite | nDer f 1 |
|  | nDer p 1 |
|  | nDer f 2 |
|  | nDer p 2 |
|  | rEur m 2 |
| Parv- | rCyp c 1 |
| albumin | rGad c 1 |
| Tropomyosin | rPen a 1 |
|  | nPen i 1 |
|  | nPen m 1 |
|  | rDer p 10 |
|  | nBla g 7 |
|  | rAni s 3 |
| Serum albumin | nBos d 6 |
|  | nFel d 2 |
|  | nCan f 3 |
|  | nEqu c 3 |
