# Supplementary material for: Food allergen sensitization pattern in adults in relation to severity of atopic dermatitis
Source: Clin Transl Allergy. 2014 Mar 28;4:9. doi: 10.1186/2045-7022-4-9 (PMC4022323; doi:10.1186/2045-7022-4-9)
Supplement: Additional file 2: Table S2 — Severity of FA by Mueller classification in patients with AD (mild/moderate versus severe AD). [file 2045-7022-4-9-S2.doc]

**Table S2 Severity of FA by Mueller classification in patients with AD
(mild/moderate versus severe AD)**

| **Group analysis** | **Total**  **n = 114 (%)** | **Mild/moderate AD**  **n = 73 (%)** | **Severe AD**  **n = 41 (%)** |
| --- | --- | --- | --- |
| **Mueller ½** | 65 (57) | 43 (59%) | 22 (54) |
| **Mueller ¾** | 49 (43 %) | 30 (41%) | 19 (46) |
| **Total** | 114 | 73 | 41 |

**p – value = 0.59;** Chi-square mild/moderate versus severe.

Allergy based on positive patient history.
