# Supplementary material for: Food allergen sensitization pattern in adults in relation to severity of atopic dermatitis
Source: Clin Transl Allergy. 2014 Mar 28;4:9. doi: 10.1186/2045-7022-4-9 (PMC4022323; doi:10.1186/2045-7022-4-9)
Supplement: Additional file 3: Table S3 — Sensitization (frequency and serum level) to food allergens of plant origin (additional allergens). [file 2045-7022-4-9-S3.doc]

**Table S3 Sensitization (frequency and serum level) to food allergens of plant origin
(additional allergens):**

| **Food** | **Allergen** |  | **Total**  **n = 211** | **Mild/moderate AD**  **n = 141** | **Severe AD**  **n = 70** | **P- value**  **n (%)^1^**  ***median^2^*** |
| --- | --- | --- | --- | --- | --- | --- |
|  |  |  |  |  |  |  |
| **Soy bean** | nGly m 4 *(PR-10)* | n (%)  n (%) *Median (IQR)* | 93 (44.1)  86 (40.8) *2.7 (1.38-8.41)* | 61 (43.3)  58 (41.1) *2.33 (1.32-4.43)* | 32 (45.7)  28 (40)  *5.83 (1.95-13.01)* | 0.74  0.88 *0.04* |
|  | nGly m 5 *(SP)* | n (%) *Median (IQR)* | 15 (7.1) *1.76 (0.59-3.9)* | 9 (6.4) *2.21 (1.75-3.57)* | 6 (8.6) *0.61(0.48-4.89)* | n.r. *n.r.* |
|  | nGly m 6 *(SP)* | n (%) *Median (IQR)* | 9 (4.3) *1.65 (1.02-3.61)* | 5 (3.5) *1.6 (0.81 -3.61)* | 4 (5.7) *2.24 (1.29-4.00)* | n.r. *n.r.* |
| **Pineappel** | nAna c 2 *(CCD)* | n (%) *Median (IQR)* | 15 (7.1) *1.31 (0.42-4.10)* | 6 (4.3) *1.33 (0.42-2.97)* | 9 (12.9) *1.08 (0.42-7.23)* | n.r.  *n.r.* |
|  |  |  |  |  |  |  |
| **Celery** | rApi g 1 *(PR-10)* | n (%) *Median (IQR)* | 54 (25.6) *2.76 (0.95-6.44)* | 37 (26.2) *2.78 (0.8-5.2)* | 17 (24.3) *2.7 (1.28-10.94)* | 0.76  *0.71* |
|  |  |  |  |  |  |  |
|  |  |  |  |  |  |  |
| **Wheat** |  | n (%) | 7 (3.3) | 3 (2.1) | 4 (5.7) | n.r. |
|  |  |  |  |  |  |  |
|  | nTri a 181 | n (%) *Median (IQR)* | 4 (1.9) *1.04 (0.59-31.45)* | 2 (1.4) *21.02 (0.47-)* | 2 (2.9) *1.04 (0.95- )* | n.r. *n.r.* |
|  | nTri a Gliadin | n (%) *Median (IQR)* | 2 (0.9)  *0.9 (0.48-)* | 1 (0.7)  0.48 | 1 (1.4)  1.31 | n.r. *n.r.* |
|  | rTri a 19.0101 | n (%) *Median (IQR)* | 0 (0) *n.r.* | 0 (0) *n.r.* | 0 (0) *n.r*. | n.r. *n.r.* |
|  | nTri a aA_TI | n (%) *Median (IQR)* | 2 (0.9)  *1.02 (0.64- )* | 0 (0) *n.r.* | 2 (2.9)  *1.02 (0.64- )* | n.r. *n.r.* |
|  |  |  |  |  |  |  |
| **Sesame** | nSes i 1 *(SP)* | n (%) *Median (IQR)* | 8 (3.8) *2.51 (0.8-15.52)* | 6 (4.3) *2.51 (1.24-9.61)* | 2 (2.9) *9.57 (0.57-)* | n.r.  *n.r.* |
|  |  |  |  |  |  |  |
| **Cashew nut** | rAna o 2 *(SP)* | n (%) *Median (IQR)* | 5 (2.4) *0.62 (0.36-0.96)* | 3 (2.1) *0.4 (0.32- )* | 2 (2.9) *0.71 (0.62- )* | n.r.  *n.r.* |
|  |  |  |  |  |  |  |
| **Brazil nut** | Ber e 1 *(SP)* | n (%) *Median (IQR)* | 12 (5.7) *1,46 (0.58-8,28)* | 4 (2.8) *2,76 (1.15-10.1)* | 8 (11.4) *1,1 (0.47 -8,3)* | n.r.  *n.r.* |

*^1^* Chi-square; mild/moderate versus severe AD; ^2^ Mann-Whitney U-test; mild/moderate versus severe AD.*** p < 0.01**; *n.r,* non-reliable, due to small number; PR-10 protein Bet v1 homulogus; SP: storage protein; LTP: lipid transfer protein; CCD: cross-reactive carbohydrate determinant
